# HTLV-1 Proviral Load in Vaginal Fluid Correlates with Levels in Peripheral Blood Mononuclear Cells

**DOI:** 10.3390/pathogens12050682

**Published:** 2023-05-05

**Authors:** Alisson de Aquino Firmino, Paulo Roberto Tavares Gomes Filho, Adenilda Lima Lopes Martins, Thessika Hialla Araújo, Luana Leandro Gois, Everton da Silva Batista, Jean Paulo Lacerda Araújo, Bernardo Galvão-Castro, Maria Fernanda Rios Grassi

**Affiliations:** 1 Integrative Multidisciplinary HTLV Center (CHTLV), Bahiana School of Medicine and Public Health (EBMSP), Salvador 40290-000, Bahia, Brazil; 2 Department of Health (DSAU), State University of Feira de Santana (UEFS), Feira de Santana 44036-900, Bahia, Brazil; 3 Department of Biointeraction Sciences, Institute of Health Sciences, Federal University of Bahia (UFBA), Salvador 40110-902, Bahia, Brazil; 4 Advanced Laboratory of Public Health (LASP), Gonçalo Moniz Institute, Oswaldo Cruz Foundation (Fiocruz), Salvador 40296-710, Bahia, Brazil

**Keywords:** HTLV-1, proviral load, vaginal proviral load

## Abstract

Background: The prevalence of human T-lymphotropic virus type-1 (HTLV-1) infection is higher in women, and sexual intercourse has been described as an important route of male-to-female transmission. The present study aimed to quantify HTLV-1 proviral load (PVL) in vaginal fluid, and to investigate correlations with PVL in peripheral blood mononuclear cells (PBMCs). In addition, cytopathological alterations and vaginal microbiota were evaluated. Methods: HTLV-1-infected women were consecutively recruited at a multidisciplinary center for HTLV patients in Salvador, Brazil. All women underwent gynecological examinations to obtain cervicovaginal fluid and venipuncture for blood collection. PVL, as measured by real-time quantitative polymerase chain reaction (RT–qPCR), was expressed as the number of copies of HTLV-1/10^6^ cells in blood and vaginal fluid samples. Light microscopy was used to assess cervicovaginal cytopathology and vaginal microbiota. Results: In the 56 included women (43 asymptomatic carriers and 13 diagnosed with HTLV-1-associated myelopathy/tropical spastic paraparesis—HAM/TSP), mean age was 35.9 (SD ± 7.2) years. PVL was higher in PBMCs (median: 23,264 copies/10^6^ cells; IQR: 6776–60,036) than in vaginal fluid (451.9 copies/10^6^ cells; IQR: 0–2490) (*p* < 0.0001). PVL in PBMCs was observed to correlate directly with PVL in vaginal fluid (R = 0.37, *p* = 0.006). PVL was detected in the vaginal fluid of 24 of 43 (55.8%) asymptomatic women compared to 12 of 13 (92.3%) HAM/TSP patients, *p* = 0.02. Cytopathologic analyses revealed no differences between women with detectable or undetectable PVL. Conclusion: HTLV-1 proviral load is detectable in vaginal fluid and correlates directly with proviral load in peripheral blood. This finding suggests that sexual transmission of HTLV-1 from females to males may occur, as well as vertical transmission, particularly in the context of vaginal delivery.

## 1. Introduction

Human T-lymphotropic virus type 1, also known as human T-leukemia virus (HTLV-1), was the first human retrovirus to be isolated and associated with disease before HIV [1]. HTLV-1 is transmitted through either i) blood, through blood transfusions in areas where screening is not performed, or through the exchange of needles and syringes containing contaminated blood; ii) from mother to child during pregnancy, at birth or mainly through breastfeeding; and iii) through unprotected sexual intercourse [2].

In the city of Salvador, Brazil, a region where HTLV-1 infection is endemic, a population-based study showed a higher prevalence of HTLV-1 infection in women that increases with age. In 2002, approximately 10% of women over 50 years of age were found to be infected with HTLV-1 [3]. In addition, sexual intercourse has been described as the main route of transmission in this city’s general population [4].

The presence of HTLV-1 has been detected in semen and cervicovaginal secretions from infected individuals [5,6]. Although a greater efficacy of male-to-female sexual transmission has been reported, female-to-male transmission also occurs and should therefore not be neglected [7,8,9,10]. HTLV-1 mainly infects CD4+ T lymphocytes, as well as CD8+ T lymphocytes to a lesser extent [11]. The virus integrates into the cell genome in the form of a provirus. Infected T cells produce few free virus particles; therefore, quantification of the integrated virus (provirus) is expressed as proviral load (PVL) [12]. In general, the proportion of lymphocytes is higher in the seminal fluid than in the vaginal fluid of healthy individuals [12], which may partly explain the greater effectiveness of male-to-female HTLV-1 transmission.

We have previously shown higher levels of Th1, Th2, and Th17 inflammatory cytokines in the vaginal fluid from HTLV-1-infected women compared to that of uninfected women [13]. In this study, we aimed to quantify PVL in the vaginal fluid of HTLV-1-infected women and then investigate correlations with PVL in peripheral blood mononuclear cells (PBMCs). In addition, cytopathological alterations and vaginal microbiota were evaluated.

## 2. Materials and Methods

### 2.1. Patients and Study Design

This was a cross-sectional observational study conducted at the Integrative Multidisciplinary HTLV Center (CHTLV) of the Bahiana School of Medicine and Public Health (EBMSP) in Salvador, Bahia, Brazil [14]. Women infected with HTLV-1 were consecutively recruited during medical consultations and referred for gynecological examinations. Relevant inclusion and exclusion criteria are described elsewhere [15]. Briefly, HTLV-1-infected women (positivity on ELISA and Western blot) aged 20 to 50 years who had been sexually active in the past four weeks were included. Patients were classified as asymptomatic (no signs of myelopathy) or diagnosed with HAM/TSP (HTLV-1-associated myelopathy/Tropical Spastic Paraparesis) according to the criteria established by Castro Costa et al. [16].

### 2.2. Ethical Approval

The present study protocol was approved by the Institutional Research Board of EBMSP (CAAE 33098414.4.0000.5544). All procedures were planned and carried out in accordance with the ethical principles established by the Declaration of Helsinki [17]. All included women signed a term of informed consent.

### 2.3. Sample Collection

Clinical and demographic data were collected using a standardized form. Whole blood samples were collected in EDTA tubes and peripheral blood mononuclear cells (PBMCs) were separated by a density gradient centrifugation and cryopreserved until use. A complete gynecologic examination and the collection of cervicovaginal specimens were performed by a single trained gynecologist [15]. In each woman, vaginal fluid was collected from the ectocervix, endocervix, and vaginal walls with a single cotton swab for PVL measurement. Samples obtained for evaluation of vaginal PVL were then placed in tubes containing 400 µL hydroxymethyl ethylene diamine tetra-acetic acid (Tris-EDTA) solution and stored at −20 °C. For cytopathological and vaginal microbiota analysis, Papanicolaou smear samples were collected from the ectocervix and endocervix using an Ayre spatula and cytobrush, respectively. The collected samples were fixed in absolute alcohol for further processing.

### 2.4. Sample Analysis

HTLV-1 PVL was measured in cervicovaginal cells and PBMCs using the same protocol. Briefly, DNA was extracted using the Spin Column DNA Extraction System (Qiagen, Hilden, Germany). The amount of cells in the vaginal fluid was not evaluated, but the extracted DNA was quantified in a UV-Vis Spectrophotometer (Nanodrop) and all samples were diluted to 30 ng/µL. HTLV-1 proviral load was quantified using a real-time TaqMan polymerase chain reaction (PCR) method, as described previously [18]. Briefly, SK110/SK111 primers were used to amplify a 186 bp fragment of the pol gene and dual TaqMan probe (5′-FAM/5′ VIC and 3′-TAMRA) was located at 4829–4858 bp of the HTLV-1 reference sequence (HTLVATK). Albumin DNA was used as an endogenous reference. The detection limit was the standard curve, which was 10^1^ copies. The value of HTLV-1 proviral load was reported as the [(HTLV-1 average copy number)/(albumin average copy number)] × 2 × 10^6^ and expressed as the number of HTLV-1 copies per 10^6^ cells in both blood and vaginal fluid samples, as previously described [13,19]. Results were analyzed using 7500 v2.0.1 software (Applied Biosystems). Cell abnormalities detected by Papanicolaou smear tests were classified in accordance with the Bethesda System using light microscopy [20].

### 2.5. Statistical Evaluations

Quantitative sociodemographic variables without normal distributions, such as family income, educational level, age of partner, number of partners and parity, are presented as medians with 25th and 75th percentiles. Age, a quantitative variable with normal distribution, is presented as mean with standard deviation. The qualitative variables skin color and marital status are expressed as simple frequencies/proportions. PVL comparisons in vaginal fluid and PBMCs were evaluated with the nonparametric Wilcoxon signed rank test, whereas comparisons between asymptomatic and HAM/TSP groups were performed with the Mann–Whitney test. Data were expressed as median values and 25th and 75th percentiles. The frequency of women with detectable PVL in vaginal fluid and PBMCs in the asymptomatic and HAM/TSP groups was evaluated using Fisher’s exact test. Spearman’s rank correlation coefficient was used to analyze the relationship between proviral load in vaginal fluid compared to PBMCs. Differences in cervicovaginal cytopathology and vaginal microbiota profiles were assessed using the Chi-squared test. *p*-values < 0.05 were considered statistically significant. All analyses were performed using GraphPad software version 9.5 and SPSS software version 17.0 for Windows.

## 3. Results

Fifty-six women were studied, 43 of whom were asymptomatic and 13 diagnosed with HAM/TSP. Almost half of the patients (46.4%) self-reported black skin color. The mean patient age was 35.9 (±7.2) years. Most participants reported being married or in a stable relationship (73.2%) with a median partner age of 42 (33.5–47.5) years. The median number of lifetime sexual partners was four (3–13.5) (Table 1).

**Figure 1:**
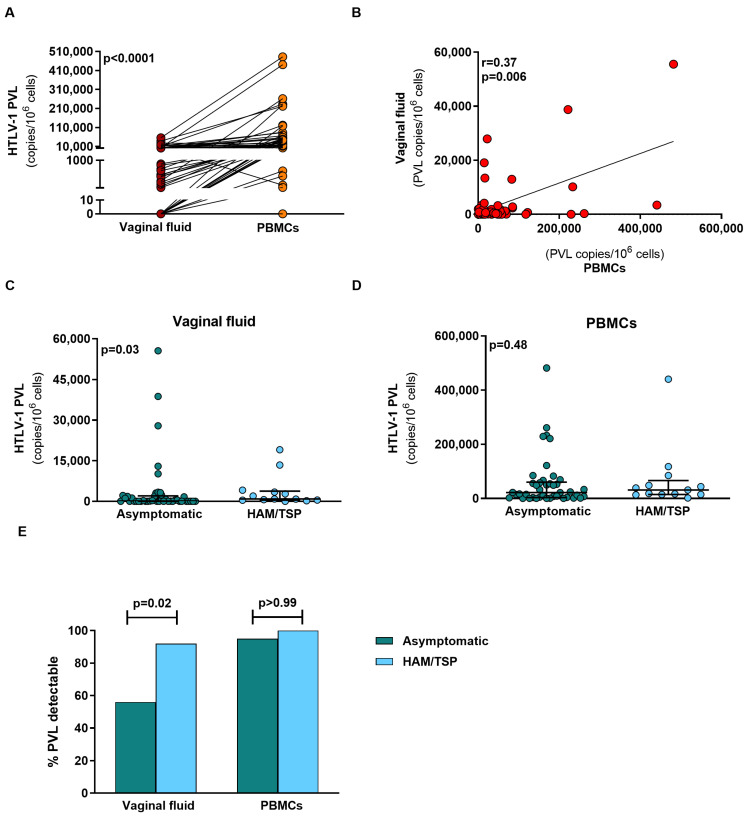
HTLV-1 proviral load (PVL) in vaginal fluid and peripheral blood mononuclear cells (PBMCs) from HTLV-1-infected women. (**A**) Comparison of median PVL in vaginal fluid and PBMCs (n = 54) using the Wilcoxon signed rank test. (**B**) Correlation between PVL in vaginal fluid and PBMCs (n = 54) using Spearman’s rank correlation testing. (**C**) HTLV-1 proviral load in vaginal fluid from asymptomatic women (n = 42) and women with HAM/TSP (n = 13) using the Mann–Whitney test. (**D**) HTLV-1 proviral load in PBMCs from asymptomatic women (n = 42) and women with HAM/TSP (n = 13) using the Mann–Whitney test. (**E**) Percentage of detectable PVL in vaginal fluid and PBMCs in asymptomatic women and women with HAM/TSP (n = 56) using Fisher’s exact test. *p*-values < 0.05 considered significant. Analysis performed using GraphPad software Prism (version 9.5, San Diego, CA, USA).

Of the 56 patients included, PVL was detectable in the vaginal fluid of 36 (64.3%) women compared to 54 women in PBMCs (96.5%). In two women, PVL was undetectable in both the vaginal fluid and PBMCs. PVL levels were statistically lower in vaginal fluid: median 451.9 copies/10^6^ cells (IQR: 0–2490), compared to PBMCs: median 23,264 copies/10^6^ (IQR: 6776–60,036) (*p* < 0.0001) (Figure 1A). A positive correlation was observed between PVL in vaginal fluid and PBMCs, R = 0.37 (*p* = 0.006) (Figure 1B).

Regarding PVL in vaginal fluid, a statistical difference was found (*p* = 0.03) between asymptomatic women (103.1 copies/10^6^ cells (IQR: 0–2000)) and carriers of HAM/TSP (869 copies/10^6^ cells (IQR: 447.9–3760)), (Figure 1C). PVL levels in PBMCs from asymptomatic carriers (21,976 copies/10^6^ cells (IQR: 3420–60,036)) were similar (*p* = 0.48) to those in patients with HAM/TSP (31,080 copies/10^6^ cells (IQR: 14,799–66,420)), (Figure 1D). PVL was detected in the vaginal fluid of 24 of 43 (55.8%) asymptomatic women compared to 12 of 13 (92.3%) HAM/TSP patients, *p* = 0.02. No significant differences were found in the PVL of PBMCs from asymptomatic women (41/43, 95.3%) and HAM/TSP patients (13/13, 100%), (Figure 1E). Two asymptomatic patients had an undetectable PVL in both vaginal fluid and PBMCs. Moreover, cytopathologic findings and vaginal microbiota were similar between these groups (Table 2).

An additional assessment of the cervicovaginal environment was performed comparing women with detectable and undetectable HTLV-1 PVL in vaginal fluid. No statistical differences were found between the groups with regard to neoplasia in the cervicovaginal cytopathology. One patient among those with detectable PVL had atypical squamous cells of undetermined significance (ASC-US). Women with undetectable PVL presented a higher frequency of *Lactobacillus* spp. (*p* = 0.004) and a lower frequency of Coccus/Bacillus (*p* = 0.001). The frequency of *Gardnerella vaginalis*/*Mobiluncus* spp. and *Candida* spp. were similar between the groups (Table 3).

## 4. Discussion

The results of the present study demonstrate a direct correlation between HTLV-1 PVL in vaginal fluid and PVL in PBMCs. Interestingly, a 50-fold difference in PVL levels were found in vaginal fluid compared to peripheral blood. Of note, PVL in vaginal fluid was undetectable in one-third of the women evaluated, almost all (n = 19/20) of whom were asymptomatic. To our knowledge, this was the first study that aimed to quantify and correlate HTLV-1 PVL in vaginal fluid and PBMCs.

The detection of HTLV-1 DNA in cervicovaginal secretion samples was previously reported in 68% of sex workers infected with the virus in Peru, which was further associated with cervicitis [21]. In addition, a study in Gabon evaluated women with HTLV-1 DNA in vaginal fluid, demonstrating the local production of anti-HTLV-1 antibodies, suggesting an immune response in the vaginal mucosa [22]. Our work has previously shown that HTLV-1-infected women exhibit an activated immune response in the vaginal mucosa, as reflected by higher concentrations of cytokines, such as IL-2, TNF, IL-10, IL-4, and IL-17, in cervical fluid compared to uninfected women. Cytokine levels in vaginal fluid have not been found to correlate with HTLV-1 PVL in cervicovaginal fluid or peripheral blood mononuclear cells in asymptomatic patients [13]. In addition, cytokine analyses revealed no differences between women with detectable or undetectable PVL (data not shown).

The role of PVL as a predictor for the development of HTLV-1-associated diseases has been suggested, as patients with HTLV-1-associated myelopathy/tropical spastic paraparesis (HAM/TSP) and adult T-cell lymphoma/leukemia (ATLL) present higher PVL in PBMCs than asymptomatic carriers [19,23]. Patients with isolated neurological changes, peripheral neuropathy, and neurogenic bladder may also have higher PVL than asymptomatic patients [24,25]. Regarding correlations between PVL in PBMCs and other body fluids, evaluation of paired breast milk and blood samples from HTLV-1-infected women showed a strong correlation between PVL in both compartments [26,27]. Furthermore, a previous study indicated a positive correlation between PVL in PBMCs and saliva, with higher mean PVL found in patients diagnosed with HAM/TSP compared to asymptomatic carriers, suggesting the possible influence of systemic inflammatory symptoms on oral health as well as potential transmission via saliva [28]. A similar correlation was found between PVL in peripheral blood and cerebrospinal fluid (CSF) by Lezin et al. in 2005, who further demonstrated a relevant association with the patients’ neurological symptoms [29]. A study conducted in Japan found that higher HTLV-1 PVL in the blood could be indicative of more severe lung involvement, which was also closely correlated with lymphocyte counts and increased PVL in bronchoalveolar fluid [30]. Thus, the presence of HTLV-1 PVL in body compartments may be directly related to localized inflammation and cytokine production, in addition to the presence of diseases associated with HTLV-1. Herein, HTLV-1 PVL was found to be much less detectable in the vaginal fluid of asymptomatic women, which correlated with lower PVL in the PBMCs from these patients. This may be due to the presence of HTLV-1-infected cells in vaginal fluid being related to the natural process of vaginal transudation, which allows lubrication through vasodilation-transudation reactions [31,32], or localized activation of the immune response in the vaginal environment. Importantly, cytopathologic findings and cytokine levels in vaginal fluid were not observed to differ between women with detectable and undetectable PVL. Moreover, cytopathologic findings were similar between asymptomatic carriers and women diagnosed with HAM/TSP. It should be noted that the PVL in the vaginal fluid could be higher than the amount detected, since quantification of PVL represents the proportion of infected cells per cell tested. Healthy vaginal fluid consists of several cell types that are not targets of HTLV-1, such as squamous cells, endocervical cells, polymorphonuclear cells, and plasmocytes [33,34]. The major targets for HTLV-1 are CD4+ T lymphocytes and, to a lesser extent, macrophages, and dendritic cells [12], which are not the predominant components of healthy vaginal fluid.

The present study, it was not possible to determine the factors for correlation of HTLV-1 PVL between vaginal fluid and PBMCs. This interference between the systemic immune response and the immune response activated in the vaginal environment could explain the higher viral concentrations of HTLV-1 in different mucous membranes. However, this still requires in-depth studies, including those on CD4+ and CD8+ T cell counts.

Regarding vaginal microbiota, significantly higher levels of *Lactobacillus* spp. were found in women with undetectable PVL in vaginal fluid, which is likely not clinically relevant considering that high levels are a common finding in the microbiota [35]. In addition, Coccus/Bacillus was more abundant in women with detectable PVL in vaginal fluid. While these pathogens may be associated with bacterial vaginosis, they are also present in healthy women [36,37]. Zunt et al. associated cervicitis with increased cervical shedding of HTLV-1 and sexual transmission [21]. However, as confirmatory testing was not performed on the present sample, it was impossible to confirm the species of pathogens found and their possible association with cervicitis, which represents a limitation regarding this association.

The presence of HTLV-1 in the vaginal fluid of infected women may have implications on the route of viral transmission. Sexual transmission of HTLV-1 can occur both from male-to-female as well as vice versa [7,8]. Overall, the frequency of CD4+ T lymphocytes, the main cells targeted by HTLV-1, is higher in the semen than in the vaginal fluid of healthy individuals [12]. However, although no studies have attempted to quantify HTLV-1 PVL in the semen of infected individuals, it has been speculated that higher PVL in semen may explain the presumed greater efficacy of male-to-female sexual transmission [5,7,8]. A report on serodiscordant couples suggested a greater risk of sexual HTLV-1 transmission to a seronegative partner when the infected partner presented higher HTLV-1 PVL in PBMCs compared to couples in which transmission did not occur [8]. On the other hand, the presence of HTLV-1 in vaginal fluid may also be relevant to the vertical transmission of HTLV-1, which is especially important considering that no consensus exists regarding the preferred route of delivery (vaginal versus cesarian) in HTLV-1-infected women. Although less common, transmission from mother-to-child occurs in up to 5% of women who have not breastfed their infants [38,39,40]. Therefore, it is of utmost importance to determine whether women with higher HTLV-1 PVL may be more likely to transmit the virus to newborns during pregnancy or delivery. To date, the use of antiretroviral drugs has not been recommended during pregnancy for HTLV-1-infected women.

## 5. Conclusions

Our results support the notion that PVL in peripheral blood may correlate with PVL in vaginal fluid, which is a relevant factor not only for the sexual route of female-to-male HTLV-1 transmission, but could also have repercussions on vertical transmission, particularly in the context of vaginal delivery.

## Figures and Tables

**Table 1:** Sociodemographic profile of 56 HTLV-1-infected women.

Variable	
Age (years) ^a^	35.9 ± 7.2
Family income ^b^	1 (1–2)
Educational level (years) ^b^	9 (5–12)
Skin color (%) ^c^	
Black	26 (46.4)
Mixed-race	21 (37.5)
White	9 (16.1)
Marital status (%) ^c^	
Married/Stable relationship	41 (73.2)
Single	14 (25)
Divorced/Separated	1 (1.8)
Partner age (years) ^b^	42 (33.5–47.5)
Number of lifetime partners ^b^	4 (3–13.5)
Parity ^b^	1.5 (1–3)

Family income is expressed as number of monthly minimum wages (USD 250 in 2023) ^a^ Data presented as mean (standard deviation). ^b^ Data presented as median and interquartile range (25th–75th percentiles). ^c^ Data presented as frequency/proportion.

**Table 2:** HTLV-1 proviral load in peripheral blood mononuclear cells and cervicovaginal fluid, cytopathologic findings, and vaginal microbiota in asymptomatic women and others with HAM/TSP.

Variable	HTLV-1 Asymptomatic (n = 43)	HAM/TSP (n = 13)	*p*-Value
Cervicovaginal cytopathology n (%) ^a^			
Negative for neoplasia	42 (97.7)	12 (92.3)	0.36
ASC-US ^b^	1 (2.3)	0	0.58
Unsatisfactory	0	1 (7.7)	0.07
Vaginal microbiota n (%) ^a^			
*Lactobacillus vaginalis*	7 (16.3)	2 (15.4)	0.94
*Gardnerella vaginalis*/*Mobiluncus* spp.	9 (20.9)	2 (15.4)	0.66
Coccus/Bacillus	16 (37.2)	6 (46.1)	0.56
*Candida* spp.	11 (25.6)	2 (15.4)	0.44
Unsatisfactory	0	1 (7.7)	0.07
HTLV-1 PVL in cervicovaginal fluid ^c,d^	103.1 (0–2000)	869 (447.9–3760)	0.03
HTLV-1 PVL in PBMCs ^c,d^	21,976 (3420–60,036)	31,080 (14,799–66,420)	0.48

^a^ Data presented as frequency/proportion; chi-square test. ^b^ Atypical squamous cells of undetermined significance (ASC-US). ^c^ Data presented (asymptomatic women (n = 42) and women with HAM/TSP (n = 13)) as median and interquartile range (25th–75th percentiles); Mann–Whitney test. ^d^ Number of HTLV-1 copies/10^6^ cells. PVL: proviral load; PBMCs: peripheral blood mononuclear cells. HAM/TSP: HTLV-1-associated myelopathy/Tropical Spastic Paraparesis.

**Table 3:** Frequency of cervicovaginal cytopathology findings and microbiota in women with detectable and undetectable proviral load in vaginal fluid.

Variable	PVL Detectable (n = 36)	PVL Undetectable (n = 20)	*p*-Value
Cervicovaginal cytopathology n (%) ^a^			
Negative for neoplasia	34 (94.4)	20 (100)	0.28
ASC-US ^b^	1 (2.8)	0	0.45
Unsatisfactory	1 (2.8)	0	0.45
Vaginal microbiota n (%) ^a^			
*Lactobacillus vaginalis*	2 (5.6)	7 (35)	0.004
*Gardnerella vaginalis*/*Mobiluncus* spp.	7 (19.4)	4 (20)	0.96
Coccus/Bacillus	20 (55.6)	2 (10)	0.001
*Candida* spp.	6 (16.6)	7 (35)	0.11
Unsatisfactory	1 (2.8)	0	0.45

^a^ Data presented as frequency/proportion; chi-square test. ^b^ Atypical squamous cells of undetermined significance (ASC-US). PVL: proviral load.

## Data Availability

The original contributions presented in the study are included in the article. Further inquiries can be directed to the corresponding author.
